# Natural small molecule FMHM inhibits lipopolysaccharide-induced inflammatory response by promoting TRAF6 degradation via K48-linked polyubiquitination

**DOI:** 10.1038/srep14715

**Published:** 2015-10-01

**Authors:** Ke-Wu Zeng, Li-Xi Liao, Hai-Ning Lv, Fang-Jiao Song, Qian Yu, Xin Dong, Jun Li, Yong Jiang, Peng-Fei Tu

**Affiliations:** 1 State Key Laboratory of Natural and Biomimetic Drugs, School of Pharmaceutical Sciences, Peking University, Beijing 100191, China; 2 Research Studio of Integration of Traditional and Western Medicine, First Hospital, Peking University, Beijing 100034, China; 3 Modern Research Center for Traditional Chinese Medicine, Beijing University of Chinese Medicine, Beijing 100029, China

## Abstract

TNF receptor-associated factor 6 (TRAF6) is a key hub protein involved in Toll-like receptor-dependent inflammatory signaling pathway, and it recruits additional proteins to form multiprotein complexes capable of activating downstream NF-κB inflammatory signaling pathway. Ubiquitin-proteasome system (UPS) plays a crucial role in various protein degradations, such as TRAF6, leading to inhibitory effects on inflammatory response and immunologic function. However, whether ubiquitination-dependent TRAF6 degradation can be used as a novel anti-inflammatory drug target still remains to be explored. FMHM, a bioactive natural small molecule compound extracted from Chinese herbal medicine Radix Polygalae, suppressed acute inflammatory response by targeting ubiquitin protein and inducing UPS-dependent TRAF6 degradation mechanism. It was found that FMHM targeted ubiquitin protein via Lys48 site directly induced Lys48 residue-linked polyubiquitination. This promoted Lys48 residue-linked polyubiquitin chain formation on TRAF6, resulting in increased TRAF6 degradation via UPS and inactivation of downstream NF-κB inflammatory pathway. Consequently, FMHM down-regulated inflammatory mediator levels in circulation, protected multiple organs against inflammatory injury *in vivo*, and prolong the survival of endotoxemia mouse models. Therefore, FMHM can serve as a novel lead compound for the development of TRAF6 scavenging agent via ubiquitination-dependent mode, which represents a promising strategy for treating inflammatory diseases.

Toll-like receptors (TLRs) in immune cells are critical for defense against invading pathogens; however, uncontrolled TLRs activation causes human inflammatory disorders and autoimmune diseases, such as endotoxemia, asthma, rheumatoid arthritis and multiple sclerosis^1^,^2^,^3^. Recognition of microbial components by TLR4 initiates downstream inflammatory events by recruiting MyD88 adaptor protein, and the formation TLR4/MyD88 signaling complex promotes the interaction of IL-1 receptor–associated kinases (IRAKs) and subsequent TNF receptor-associated factor (TRAF6) and TGF-β activated kinase 1 (TAK1), further leading to the activation and nuclear translocation of NF-κB transcription factor^4^,^5^, as well as increased expressions of proinflammatory cytokines, such as TNF-α, IL-6 and IL-1β^6^. Since over-activation of TLR4/TRAF6/NF-κB signaling pathway is often associated with inflammatory diseases; precise regulation must be maintained at cellular levels for our body homeostasis^7^,^8^.

Protein ubiquitination is a crucial post transcriptional modification to regulate the intensity of cell signaling pathway. Lysine 48 (K48)-linked protein ubiquitination is responsible for protein degradation by the ubiquitin-proteasome system^9^,^10^. Meanwhile, unlike K48, Lysine 63 (K63)-linked protein ubiquitination does not target the proteins for proteasomal degradation, but instead serves as a scaffold to connect the upstream and downstream signaling proteins^11^. As a hub of inflammatory signaling, TRAF6 is an essential adaptor protein for TLR4, and modification by ubiquitination on TRAF6 can directly control the fortune of TLR4-dependent inflammatory pathway^12^. Normally, TRAF6 forms a homodimer and catalyzes K63-linked ubiquitination on itself. K63-linked ubiquitination of TRAF6 is essential for the activation of TLR4-dependent NF-κB signaling through supporting an anchoring platform for corresponding downstream adaptor proteins such as TAK1 and NEMO, further leading to the phosphorylation and nuclear translocation of NF-κB transcription factor^13^. Interestingly, recently it is found that TRAF6 can also be modified by K48-linked ubiquitination, which can promote TRAF6 degradation through proteasome and block the inflammatory signal transduction in TLR4 pathway^14^,^15^,^16^,^17^. Therefore, alternation of the balance between the K48 and K63-linked ubiquitination could trigger a cascade response towards either anti-inflammation or pro-inflammation. The promotion of the assembly of K48-linked polyubiquitination chains on TRAF6 becomes a promising strategy to negatively regulate TLR4-mediated NF-κB signaling pathway and suppress excessive inflammatory responses. However, there has not been any candidate drug targeting on promoting TRAF6 degradation via K48-linked ubiquitination. Thus, aim to alter the central protein stability involved in TLR4-NF-κB pathway, such as TRAF6, offering an unprecedented strategy to treat inflammatory diseases.

Natural products have played a major role in discovering novel drugs for the pathobiology of infection and inflammatory diseases^18^,^19^. Bioactive small molecules from medicinal herbs form a treasury that supplies candidate drugs with structure-function diversity^20^. Prior to the present study, it was unclear whether a natural small molecule could induce TRAF6 degradation through mediating protein post-translational modification via specific polyubiquitin chain editing. A natural small molecule extracted from a traditional Chinese herbal medicine Radix Polygalae, (5-formylfuran-2-yl) methyl 4-hydroxy-2-methylenebutanoate (FMHM), has been previously found to show inhibitory effects on microglia activation via suppression of aldose reductase and protein kinase c^21^. The present study explored the inflammation-regulating mechanism of FMHM by activity-based protein profiling technology (ABPP) and identified FMHM as a TRAF6 degrading agent via specifically editing polyubiquitin chains.

## Results

### FMHM inhibits the production of inflammatory mediators

**Figure 1:**
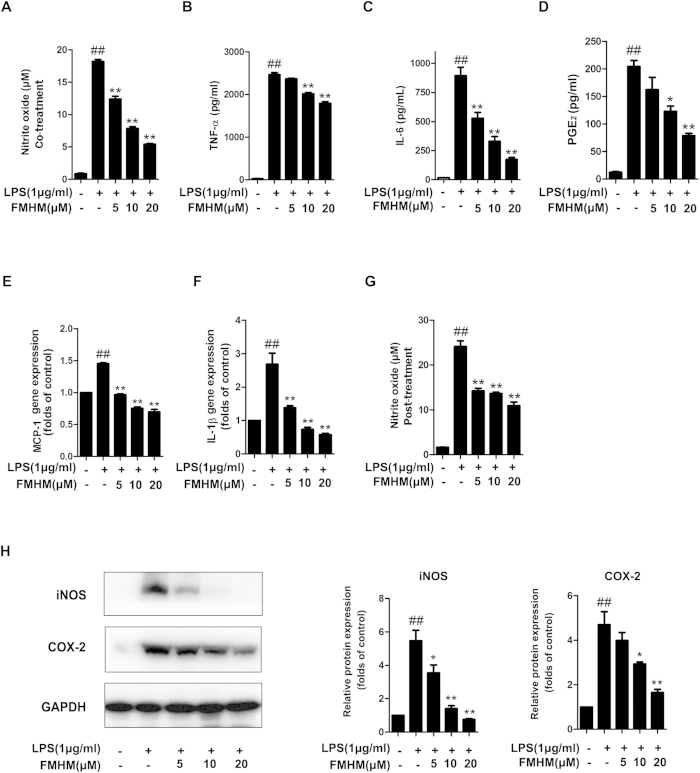
FMHM inhibits the production of inflammatory mediators. RAW264.7 cells (**A–D**) were treated with LPS (1 μg/mL) in the absence or presence of FMHM (5, 10 and 20 μM) for 4 h (TNF-α), 8 h (IL-6) and 24 h (NO and PGE_2_), and then the releases of NO, TNF-α, IL-6 and PGE_2_ were detected by Griess and ELISA assay. (**E,F**) RAW264.7 cells were treated with LPS (1 μg/mL) in the absence or presence of FMHM (5, 10 and 20 μM) for 6 h. The mRNA expressions of MCP-1 and IL-1β were detected by real-time PCR analysis. (**G**) RAW264.7 cells were treated with LPS (1 μg/mL) for 1 h, followed by the treatment with or without FMHM (5, 10 and 20 μM) for 24 h. The release of NO was detected by Griess assay. (**H**) RAW264.7 cells were treated with LPS (1 μg/ml) in the absence or presence of FMHM (5, 10 and 20 μM) for 24 h, and then Western blotting assay was performed to detect iNOS and COX-2 proteins. All data are presented as means ± S.D. from independent experiments performed in triplicate. ^##^*P* < 0.01, relative to control group; ^*^P < 0.05, ^**^P < 0.01, relative to LPS group.

In the previous activity screening experiment, we identified FMHM as a potential anti-inflammatory compound. Therefore, the present study firstly confirmed the *in vitro* inhibitory effect of FMHM on LPS-induced inflammation using a mouse macrophage-like cell line (RAW264.7). Figure 1A–D showed that 5–20 μM FMHM significantly decreased the releases of NO, TNF-α, IL-6 and PGE_2_ in a concentration-dependent manner. Moreover, FMHM suppressed the gene expressions of MCP-1 and IL-1β (Fig. 1E,F), indicating a marked inhibitory effect on inflammatory response *in vitro*. Interestingly, post-treatment with FMHM still exerted obviously inhibitory effects on NO production in LPS-induced RAW264.7 cells (Fig. 1G). Immunoblotting assay revealed that FMHM also effectively inhibited some pivotal inflammatory protein expressions such as inducible nitric oxide synthase (iNOS) and cyclooxygenase (COX-2) (Fig. 1H). Furthermore, cell viability assay indicated that 5–20 μM FMHM was not cytotoxic (Supplement Figure S1), excluding the possibility that the observed inflammatory inhibition effects might be resulted from FMHM-induced cytotoxicity. Taken together, these results suggested that FMHM is a potent anti-inflammatory active compound which can be used as a therapeutic compound against inflammatory diseases.

### FMHM inhibits inflammation by inactivating TLR4/MyD88-dependent NF-κB pathway

To explore whether FMHM could interfere the binding of LPS to cell surface, RAW264.7 cells were incubated with Alexa Fluor-488 conjugated-LPS with or without FMHM, and the fluorescence intensity of cells was analyzed by flow cytometry. Results showed that FMHM did not change the fluorescence intensity of Alexa Fluor-488 conjugate-LPS on the surface of RAW264.7 cells, suggesting FMHM did not disturb the binding of LPS to cell surface (Supplement Figure S2). Moreover, ciclosporin A was used (CsA, P-glycoprotein Pgp inhibitor^22^) to treat the cells to block the drug efflux function of Pgp, and then we observed the inflammatory inhibitory effects of FMHM were enhanced compared with non-CsA treated cells (Supplement Figure S3). These results suggested that FMHM targets intracellular proteins but not the proteins anchored in cell surface, because CsA treatment could inhibit FMHM efflux and increase intracellular FMHM concentration as well as its biological effects.

**Figure 2:**
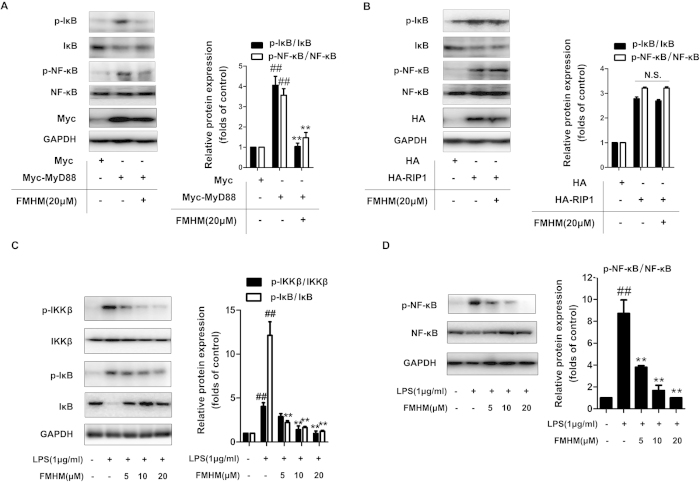
FMHM inhibits inflammation by inactivating TLR4/MyD88-dependent NF-κB pathway. (**A**) RAW264.7 cells were transfected with Myc-tagged MyD88 plasmids for 72 h, and then treated with LPS (1 μg/mL) in the absence or presence of FMHM (20 μM) for 1 h. (**B**) RAW264.7 cells were transfected with HA-tagged RIP1 plasmids for 72 h, and then  treated with LPS (1 μg/mL) in the absence or presence of FMHM (20 μM) for 1 h. (**C**,**D**) RAW264.7 cells were treated with LPS (1 μg/mL) in the absence or presence of FMHM (5, 10 and 20 μM) for 1 h. Western blotting assay was performed to detect of phosphorylation and total of IKKβ, IκB and NF-κB protein expressions. All data are presented as means ± S.D. from independent experiments performed in triplicate. ^##^*P* < 0.01, relative to control group; ^**^P < 0.01, relative to LPS or MyD88 transfection group.

TLR4 signal is a crucial transduction pathway for LPS-induced systemic inflammation^23^. The binding of LPS to TLR4 recruits adaptor proteins to the cytoplasm domain of TLR4 and activates downstream NF-κB pathway via MyD88-dependent and receptor-interacting protein (RIP1)-dependent manners, leading to the major inflammatory gene transcriptional activation^24^,^25^. In order to identify which pathway (TLR4/MyD88-dependent or TLR4/RIP1-dependent) was involved in FMHM-mediated anti-inflammation signal, we established specific MyD88 and RIP1-dependent NF-κB activation cell models by transfecting MyD88 and RIP1-high expression plasmids into RAW264.7 cells, separately. As shown in Fig. 2A, the phosphorylation levels of IκB and NF-κB were significantly increased in MyD88-transfected cells, showing that NF-κB pathway was activated. Treatment with FMHM significantly inhibited the phosphorylation of IκB and NF-κB in MyD88-transfected cells. Moreover, the phosphorylation levels of IκB and NF-κB were also significantly increased in RIP1-transfected cells; however, FMHM failed to induce any decrease of IκB or NF-κB phosphorylation levels, suggesting that FMHM inhibited NF-κB mainly via MyD88-dependent signaling pathway, rather than RIP1-dependent one (Fig. 2B).

Moreover, in non-transfected RAW264.7 cells, it was observed that FMHM inhibited the phosphorylations of IκB kinase (IKKβ), IκB and NF-κB in a concentration-dependent manner (Fig. 2C,D), further verifying that FMHM blocked LPS/TLR4 signal activation via MyD88, and led to the suppression of downstream IKK-IκB-NF-κB pathway. Besides TLR4/MyD88-dependent canonical NF-κB pathway, we also tested the regulatory effects of FMHM on non-canonical inflammatory pathway including TRAF2, TRAF3, NIK, RelB and p52, the mature form of NF-κB2^26^. It was found that FMHM treatment did not show any effect on the expression levels of these proteins (Supplement Figure S4). These results indicate that FMHM inhibits inflammatory response by selectively blocking the TLR4/MyD88-dependent canonical NF-κB pathway.

### FMHM down-regulates TRAF6 expression via ubiquitin-dependent protein degradation mode

**Figure 3:**
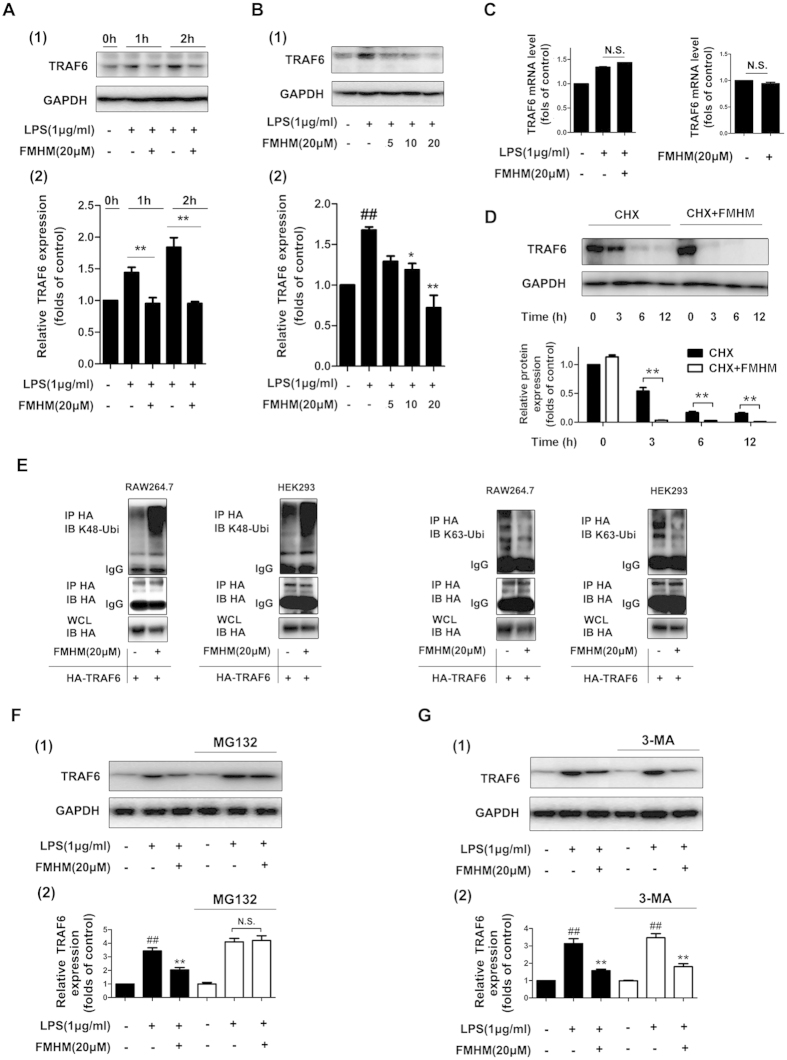
FMHM down-regulates TRAF6 expression via ubiquitin-dependent protein degradation mode. (**A**) RAW264.7 cells were treated with LPS (1 μg/mL) in the absence or presence of FMHM (20 μM) for 1 and 2 h. Western blot assay was performed for detection of TRAF6 protein expressions. (**B**) RAW264.7 cells were treated with LPS (1 μg/mL) in the absence or presence of FMHM (5, 10 and 20 μM) for 1 h. Western blotting assay was performed to detect TRAF6 protein. (**C**) RAW264.7 cells were treated with FMHM (20 μM) in the absence or presence of LPS (1 μg/mL) for 6 h. Real-time PCR was performed to detect TRAF6 mRNA expression. (**D**) RAW264.7 cells were treated with cycloheximide (50 μM) alone or together with FMHM (20 μM) for 0, 3, 6 and 12 h. Western blotting assay was performed to detect TRAF6 protein. (**E**) RAW264.7 and HEK293T cells were transfected with HA-tagged TRAF6 plasmids for 72 h, and then treated with FMHM (20 μM) for 1 h. Co-IP assay was performed to detect different types of ubiquitination (K48-linked and K63-linked) levels of TRAF6. (**F**) RAW264.7 cells were pretreated with or without MG132 (20 μM) for 4 h, and further treated with FMHM (20 μM) in the absence or presence of LPS (1 μg/mL) for 1 h. Western blotting assay was performed to detect TRAF6 protein expressions. (**G**) RAW264.7 cells were pretreated with or without 3-MA (2 mM) for 4 h, and further treated with FMHM (20 μM) in the absence or presence of LPS (1 μg/mL) for 1 h. Western blotting assay was performed to detect TRAF6 protein. All data are presented as means ± S.D. from independent experiments performed in triplicate. ^##^*P* < 0.01, relative to control group; ^**^P < 0.01, relative to LPS group.

TRAF6 is a crucial hub protein for linking TLR4/MyD88 complex and downstream NF-κB inflammatory pathway^27^. Since FMHM did not alter the expression of TLR4 or MyD88 (Supplement Figure S5), we further tested TRAF6 expression level by Western blotting assay upon FMHM treatment. The results demonstrated that FMHM down-regulated TRAF6 protein expression in a dose-dependent manner (Fig. 3A,B) without affecting the mRNA level of TRAF6 (Fig. 3C). Moreover, we used cycloheximide (protein synthesis inhibitor) to inhibit TRAF6 protein synthesis, and it was found that FMHM also significantly decreased TRAF6 protein level in a time-dependent manner, indicating that FMHM did not regulate protein translation process, but influenced the post-translational modification of TRAF6 (Fig. 3D).

The most important protein degrading mode in post-translational modification is ubiquitination^28^. Therefore, we purified HA-tagged TRAF6 proteins by immunoprecipitation from HA-TRAF6 transfected RAW264.7 and HEK293T cells with or without FMHM treatment, and detected the K48-specific and K63-specific polyubiquitin levels. As shown in Fig. 3E, FMHM obviously increased the K48-specific polyubiquitin expression on TRAF6; however FMHM decreased K63-specific polyubiquitin expression on TRAF6 in both two cell types. Because K48-linked ubiquitination was involved in protein degradation process through proteasome system, these observations suggested that FMHM might promote TRAF6 degradation via induction of K48-specific polyubiquitin formation on TRAF6. Additionally, we found that FMHM treatment still could markedly induce K48-linked ubiquitination of TRAF6 in the presence of MG132. This indicated that FMHM promoted TRAF6 degradation mainly by mediating K48-linked ubiquitination of TRAF6, but not on proteasome (Supplement Figure S6). Here, we also found that FMHM treatment induced higher overall ubiquitination level than untreated cells. Thus, we speculated that the higher ubiquitination level of TRAF6 might be a reflection of global ubiquitination status of TRAF6, and most of which might be resulted from K48-linked ubiquitination. Of course, this needs further evidence in the future (Supplement Figure S7).

To further verify this result, we used MG132 (proteasome inhibitor) to inactivate ubiquitin-proteasome system and found that FMHM did not induce TRAF6 protein degradation (Fig. 3F). It is worth mentioning that autophagy is also a major cell process involved in protein degradation besides ubiquitination. Therefore, we used 3-MA (autophagy inhibitor) to treat the cells and explored the regulatory effects of FMHM on TRAF6. Figure 3G showed that FMHM could markedly down-regulate TRAF6 level under 3-MA treatment, indicating that autophagy is not a major process involved in FMHM-mediated TRAF6 degradation. Additionally, this observation was also verified by another TRAF6 degradation experiment using autophagy inhibitor Bafilomycin A1 in our research (Supplement Figure S8).

### Ubiquitin was identified as the target protein of FMHM

**Figure 4:**
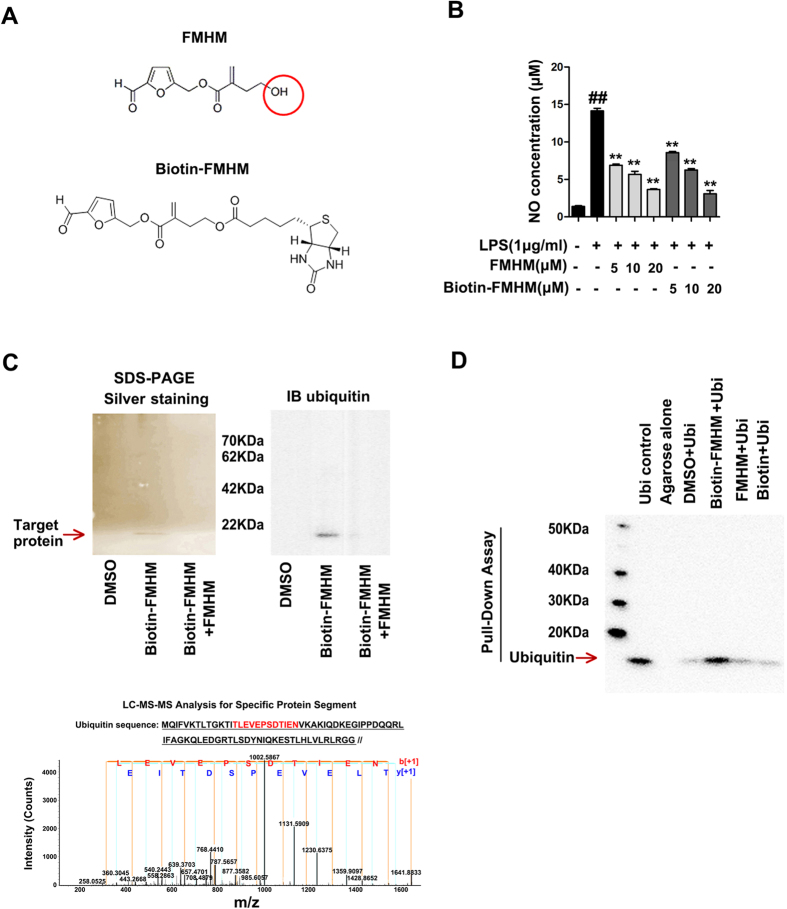
Ubiquitin was identified as the target protein of FMHM. (**A**) Structure of biotin-labeled FMHM (Bio-FMHM). (**B**) RAW264.7 cells were treated with LPS (1 μg/mL) in the absence or presence of FMHM (5, 10 and 20 μM) and Bio-FMHM (5, 10 and 20 μM) for 24 h, and then the release of NO was detected. (**C**) Bio-FMHM was incubated with cell lyses for 2 h and FMHM-associated protein was isolated with Avidin-agarose beads by immunoprecipitation (IP). Protein precipitation was separated by SDS-PAGE and stained by silver staining and Western blotting. (**D**) Recombinant ubiquitin protein was incubated with biotin (20 μM), Bio-FMHM (20 μM) and FMHM (20 μM). After 2 h at 4 °C, the mixtures were incubated with Avidin-agarose and shaken for 1 h at room temperature. The elution was separated and detected by Western blotting assay. All data are presented as means ± S.D. from independent experiments performed in triplicate. ^##^*P* < 0.01, relative to control group; ^**^P < 0.01, relative to LPS group.

We next searched for the potential targets of FMHM that caused TRAF6 degradation and anti-inflammatory action. Thus, biotin was used as a useful chemical probe to label FMHM (Fig. 4A)^29^. In order not to influence the structure-activity basis of FMHM, we firstly compared the anti-inflammatory activity of Bio-FMHM and FMHM on LPS-induced NO production model. From Fig. 4B, both FMHM and Bio-FMHM showed significant inhibitory effects on NO production in a concentration-dependent manner, indicating that biotin label did not significantly affect the pharmacological features of FMHM. Furthermore, we applied activity-based protein profiling (ABPP) technology to identify the target of FMHM^30^. RAW264.7 whole cell lysates were incubated with Bio-FMHM or together with excess FMHM (10 times of Bio-FMHM) as a competition group. Then, the compound-target protein complexes were precipitated with streptavidin-coated agarose beads by specific biotin-streptavidin interaction, followed by SDS-PAGE and silver staining. We found only one small protein band (molecular mass less than 10 kDa) was precipitated by Bio-FMHM but not by FMHM competition group (Fig. 4C), suggesting that the protein bound to Bio-FMHM also bound to FMHM. The protein band was analyzed by LC-MS/MS and 6 unique peptides for ubiquitin (Ub) protein were identified, including TITLEVEPSDTIENVK; IQDKEGIPPDQQR; AKIQDKEGIPPDQQR; LIFAGKQLEDGR; MQIFVKTLTGK; QLEDGRTLSDYNIQK. Moreover, the protein sequence coverage was 83.58%. Therefore, the FMHM-bound protein was identified as ubiquitin protein (Fig. 4C). This observation was also confirmed by Western blotting using specific anti-ubiquitin antibody (Fig. 4C). To confirm that FMHM did form complex with ubiquitin, we then used streptavidin-coated agarose beads to pull down the possible protein complex of Bio-FMHM/recombinant ubiquitin protein, and performed Western blotting for specific anti-ubiquitin antibody. We found that Bio-FMHM group showed significant ubiquitin band (Fig. 4D), suggesting that Bio-FMHM strongly associated with ubiquitin. In contrast, in DMSO, FMHM and Biotin groups (as negative controls), the ubiquitin bands were very weak, indicating that the binding of Bio-FMHM with ubiquitin was specific. Taken together, these observations confirmed that FMHM could specifically bind with ubiquitin.

### FMHM promotes ubiquitination-dependent TRAF6 degradation by binding to lysine residue on ubiquitin and inhibits ubiquitin-E2 thioester bond formation

**Figure 5:**
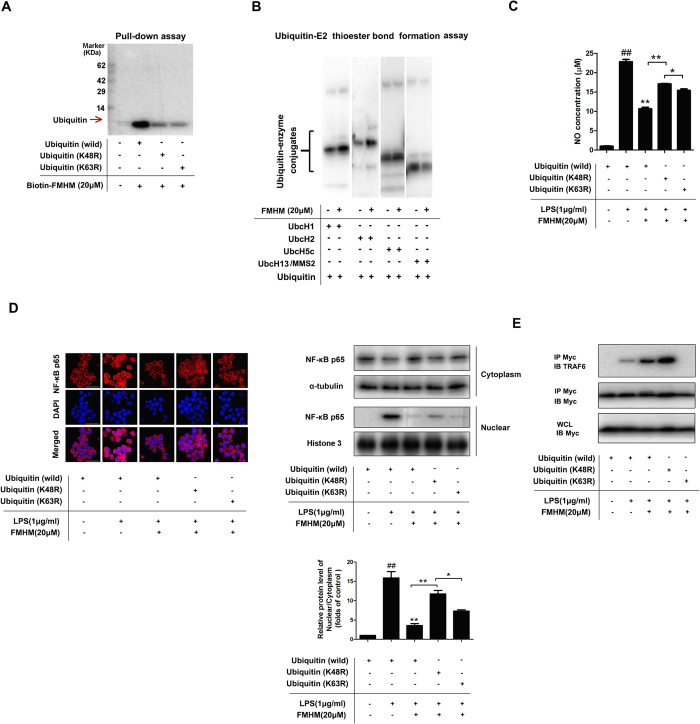
FMHM promotes ubiquitination-dependent TRAF6 degradation by binding to lysine residue on ubiquitin and inhibits ubiquitin-E2 thioester bond formation. (**A**) Recombinant ubiquitin protein (wild-type, K48R mutant, K63R mutant) were incubated with Bio-FMHM (20 μM) at 4 °C for 2 h, followed by pull-down analysis with Avidin-agarose beads for the reaction samples and silver staining. (**B**) The loading of ubiquitin (Ub) to E2 enzyme was investigated by mixing below components together at 37 °C for 60 minutes, including purified recombinant E1 Enzyme-UBE1,Ub, and E2 enzymes including UbcH1, UbcH2, UbcH5c, UbcH13/MMS2, Ubiquitinylation Buffer, DTT, MgCl_2_ as described in methods section. Then, the samples were detected by Western blot analysis. (**C**) RAW264.7 cells were transfected with Myc-tagged ubiquitin plasmids (wild-type, K48R or K63R mutant) for 72 h, and then treated with LPS (1 μg/mL) in the absence or presence of FMHM (20 μM) for 24 h Then, the release of NO was detected. (**D**) RAW264.7 cells were transfected with Myc-tagged ubiquitin plasmids (wild-type, K48R or K63R mutant) for 72 h, and then treated with LPS (1 μg/mL) in the absence or presence of FMHM (20 μM) for 1 h. Then, the nuclear translocation of NF-κB was detected by immunofluorescence assay and western blot assay using specific anti-NF-κB p65 antibody. (**E**) RAW264.7 cells were transfected with Myc-tagged ubiquitin plasmids (wild-type, K48R, K63R mutation) for 72 h, and then treated with LPS (1 μg/mL) in the absence or presence of FMHM (20 μM) for 1 h. Co-IP assay was performed to detect the amounts of ubiquitin-modified TRAF6. All data are presented as means ± S.D. from independent experiments performed in triplicate. ^##^*P* < 0.01, relative to control group; ^**^P < 0.01, relative to LPS group.

Since K48 and K63 were two vital sites for polyubiquitin chain formation, we then mainly investigated the possible action mode of FMHM on these sites^31^,^32^. To explore the potential residue of ubiquitin that interacted with FMHM, we incubated the recombinant ubiquitin proteins (wild-type, K48R mutant, K63R mutant) with Bio-FMHM, followed by pull-down analysis. We found that Bio-FMHM strongly bound to ubiquitin (wild-type), which was shown by one obvious specific band in the gel (Fig. 5A). In contrast, after incubation with K48R or K63R mutated ubiquitin, the bands were weakened, suggesting that Bio-FMHM may associate with ubiquitin by targeting K48 and/or K63 sites. Thereby, our results suggested that K48 and K63 residues were likely to be the residues involved in the direct interaction with FMHM. Next, we performed ubiquitin-E2 thioester bond formation assay *in vitro* to investigate which types of polyubiquitin chain (K48-linked and K63-linked) formation were regulated by FMHM. We found that FMHM markedly increased the thioester bond formation of UbcH1, UbcH2, UbcH5c, which mainly catalyses the synthesis of polyubiquitin chains linked via K48, and inhibited the thioester bond formation of UbcH13/MMS2, which mainly catalyses the synthesis of polyubiquitin chains linked via K63 (Fig. 5B).

Moreover, we used NO production model to investigate whether the anti-inflammatory effects of FMHM were regulated by different amino residues on ubiquitination. FMHM significantly inhibited NO production in wild-type -ubiquitin-transfected RAW264.7 cells; however, this effect was reversed in RAW264.7 cells transfected with either K48R mutated or K63R mutated ubiquitin (Fig. 5C). Thus, we proposed that K48R mutation destroyed the FMHM-targeted handle in ubiquitin and blocked the formation of K48-linked polyubiquitin chain on TRAF6, leading to the suppression of TRAF6 degradation and activation of NF-κB inflammatory pathway. Interestingly, this speculation was supported by NF-κB immune staining assay. FMHM markedly inhibited NF-κB nuclear translocation in wild-type-ubiquitin-transfected RAW264.7 cells stimulated with LPS (Fig. 5D); however, this inhibitory effect was blocked in K48R or K63R mutated ubiquitin-transfected RAW264.7 cells. Therefore, both K48R and K63R mutation in ubiquitin abolished FMHM-mediated NF-κB inactivating action, suggesting that FMHM might inhibit NF-κB inflammatory pathway by interacting with ubiquitin and promoting polyubiquitin chain formation on TRAF6 as well as ubiquitin-dependent TRAF6 degradation. Here, the K48R and K63R mutant cells looked the same as wild-type cells, at least in specific NF-κB immunofluorescence. Therefore, the transfection process did not affect our observation on NF-κB translocation and activation upon different drug treatments. Finally, we transfected RAW264.7 cells with Myc-tagged ubiquitin plasmids (wild-type, K48R mutant and K63R mutant) and treated the cells with LPS in the presence and absence of FMHM. Immunoprecipitation assay with anti-Myc antibody revealed that FMHM could induce obvious increase of TRAF6 binding with wild-type ubiquitin. Moreover, K48R mutation markedly enhanced the intensity of TRAF6 specific band. This may be resulted from that K48-linked polyubiquitin by FMHM was blocked in K48R mutation cells, and ubiquitin-dependent TRAF6 degradation was stopped; therefore, TRAF6 accumulated in cells, leading to the high expression of monoubiquitin-modified TRAF6 (Fig. 5E). However, TRAF6 band became much weaker in cells transfected with K63R mutated ubiquitin, we speculated that K63R mutation inhibited the binding of FMHM to ubiquitin via K63 site, which indirectly caused more FMHM association with ubiquitin via K48, finally leading to TRAF6 degradation through ubiquitination (Fig. 5E).

### FMHM exerts anti-inflammatory effects *in vivo*

**Figure 6:**
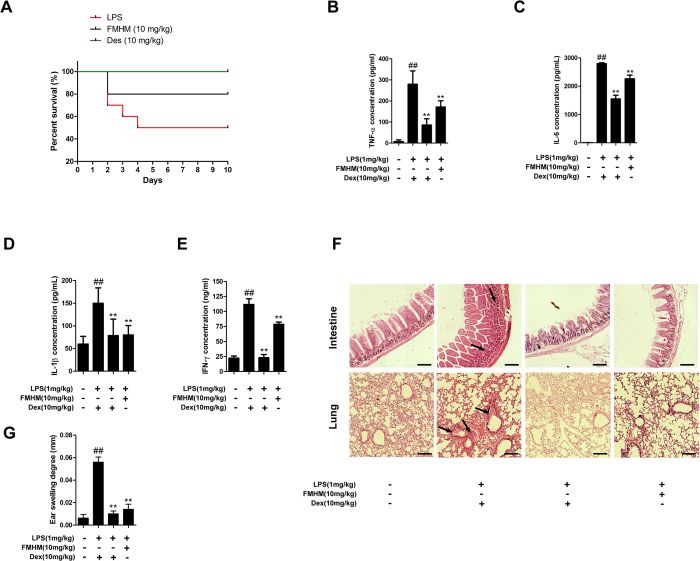
FMHM exerts anti-inflammatory effects *in vivo*. (**A**) For survival curve analysis, Balb/c mice received intraperitoneal injection of FMHM (10 mg/kg) for 1 h, and then followed with intraperitoneal injection of LPS (1 mg/kg). Animals were continuously observed and the number of survival mice was documented for 10 consecutive days. Then, the survival curve was drawn with software GraphPad Prism 5. (**B–E**) Balb/c mice received intraperitoneal injection of FMHM (10 mg/kg) for 1 h, and then followed with intraperitoneal injection of LPS (1 mg/kg) for 3 h. The bloods were collected and inflammatory mediators in serum were detected by ELISA assay. (**F**) Lungs and intensities were removed and hematoxylin-eosin staining (HE) assay was performed. Arrows indicated typical inflammatory cells infiltration. (**G**) Balb/c mice received intraperitoneal injection of FMHM (10 mg/kg) for 1h, and then xylene was painted on one ear of each animal for 30 min. The thickness of each pair of ears was measured and the difference values of each pair of ears were calculated. Dexamethasone (10 mg/kg) was used as positive control. All data are presented as means ± S.D. from independent experiments performed in triplicate. ^##^*P* < 0.01, relative to control group; ^**^P < 0.01, relative to LPS group.

To evaluate the therapeutic value of FMHM for inflammatory diseases, we investigated its *in vivo* anti-inflammatory effects in a mouse model of endotoxemia induced by LPS. Following LPS stimulation, about half mice died within 4 days, whereas, only 20% of the FMHM-treated mice died within 4 days (Fig. 6A). FMHM also remarkably decreased the circulation levels of pro-inflammatory mediators such as TNF-α, IL-6, IL-1β and IFN-γ in LPS-challenged mice (Fig. 6B–E). Moreover, FMHM protected mice from LPS-induced intestine and lung damage as shown in Fig. 6F. The inflammatory infiltration in these tissues was effectively suppressed by FMHM treatment, leading to the decreasing number of macrophage aggregates. Furthermore, the anti-inflammatory effects of FMHM were assessed by xylene-induced ear swelling model. Xylene induced significant ear swelling compared with the control model (Fig. 6G); however, FMHM treatment significantly ameliorated this pathological process by inhibiting the swelling degree. These data confirmed that FMHM was able to protect the mice from mild and severe inflammatory injury and prolong their survival time.

## Discussion

Inflammatory response is a fundamental character of many complicated diseases. In fact, multi-pathogenesis of human major diseases such as atherosclerosis, diabetes, and aging-related neurological diseases are all closely linked with inflammatory status. Previous reports showed that endotoxins played a key role in the pathogenesis of inflammation especially for endotoxemia in patients^33^. Increasing evidence suggests that chronic infection, obesity, alcohol consumption and aging can contribute to the rise of circulating endotoxin levels and cause low-grade chronic endotoxemia. Moreover, this low-grade endotoxemia may further promote internal immune environment into a pro-inflammatory state if not being controlled properly, eventually eliciting the pathogenesis of inflammatory diseases^34^. Thus, effective control of endotoxemia in the beginning could avoid or at least delay inflammation-related diseases. Here we reported a natural small molecule FMHM which inhibited inflammatory mediator releases in bacterial endotoxin (LPS)-induced *in vitro* and *in vivo* inflammatory models, and protected multiple organ injuries against inflammatory responses with prolonged survival in a mouse model of acute endotoxemia.

Activity-based protein profiling technology (ABPP) is promising strategy for identifying direct protein target of small molecule drug^34^,^35^. We modified the molecule structure of FMHM by linking the biotin tag to its hydroxide radical (Bio-FMHM) and identified the target protein of FMHM as ubiquitin using biotin-tag affinity purification via avidin-biotin interaction. It is worth noting that, in order not to influence the structure-activity basis of FMHM, we compared the anti-inflammatory activity of Bio-FMHM derivative and FMHM on LPS-induced NO production model. We observed that NO inhibitory activity did not change between Bio-FMHM and FMHM, suggesting that hydroxide radical was not the pharmacophore of FMHM and Bio-FMHM could be used as reasonable probe for fishing the target protein of FMHM.

Ubiquitin-proteasome system (UPS) has been reported to play a crucial role in TNF receptor-associated factor 6 (TRAF6) degradations, leading to inhibitory effects on inflammatory response^14^,^15^,^16^,^17^. Here, we found that FMHM might target K48 residue in ubiquitin and promote K48-linked polyubiquitin chain formation on TRAF6. This may result in more TRAF6 degradation via ubiquitin-proteasome system which was observed previously. Interestingly, unlike K48, FMHM decreased the K63-specific polyubiquitin chain formation on TRAF6. It was hypothesized that the interaction modes of FMHM with two sites seemed to be different from one another. Nevertheless, this conjecture needs further investigation on protein-small molecule co-crystallization analysis. It is worth noting that cysteine residue in proteins is usually identified as a crucial drug-targeting site. The sulfhydryl group in cysteine is inclined to attack unsaturated structure in drug compound to form covalent bonds^35^. Taken together, FMHM-induced K48-linked polyubiquitin chain formation and subsequent degradation of TRAF6 may explain the major anti-inflammation mechanism of FMHM.

Here, the most worthwhile point is that E2s are less specific than E3 ligases in most cases. Therefore, the possibility that FMHM might affect E3 ligases cannot be excluded. Interestingly, we found several previous studies have reported that TRAF6 ubiquitination could be mediated by E3 ligases. First, TRAF6 has an E3-ubiquitin ligase activity and can regulate the ubiquitination of TRAF6 by itself 36. Secondly, several E3-ubiquitin ligase which could regulate TRAF6 ubiquitination have also been discovered recently. For example, E3-ubiquitin ligase Pellino3 can negatively mediate TRAF6 ubiquitination^37^; ubiquitin E3 ligase Itch interacts with the deubiquitinating enzyme and is important for TRAF6 deubiquitination^38^; furthermore, the WW domain containing E3 ubiquitin protein ligase 1 (WWP1) acts as an E3 ligase upon LPS stimulation, and can bind to TRAF6 and promote K48-linked polyubiquitination on TRAF6^16^. Therefore, we speculated that E3 ligase activity on ubiquitinating or deubiquitinating TRAF6 might be also affected by FMHM and further verification should be certainly done in the future.

In conclusion, considering the pivotal roles of NF-κB in immune responses to pathogens, it is plausible that the ubiquitination-dependent of TRAF6 degradation is a promising drug target for inflammation diseases and FMHM is a novel anti-inflammation drug candidate for this target. Future research will aim at developing improved FMHM analogs that are more potent for TRAF6 degradation and inflammation suppression.

## Methods

### Materials

Compound FMHM (C_11_H_12_O_5_, molecular weight 224) was obtained from the Department of Natural Medicinal Chemistry, School of Pharmaceutical Sciences, Peking University Health Science Center^39^. High-performance liquid chromatography showed the purity >98%. Alexa Fluor 488 donkey anti-mouse IgG (H+L) and DyLight 594 Goat anti-rabbit IgG (H+L) were purchased from Invitrogen (Carlsbad, CA, USA). Fetal bovine serum (FBS), Dulbecco’s Modified Eagle medium (DMEM), antibiotics, and trypsin were from Hyclone (MA, USA). Lipopolysaccharide (LPS; from *Escherichia coli*, serotype 055:B5) was purchased from Sigma-Aldrich (St Louis, MO, USA). Primary antibodies were purchased from Cell Signaling Technology and Abcam (Supporting information for antibody). Western Chemiluminescent HRP substrate was obtained from Pierce Scientific (Rockford, IL, USA). PGE_2_ ELISA kit and recombinant ubiquitin (wild-type, K48R mutation, K63R mutation) were from R&D systems (Minneapolis, MN, USA). TNF-α, IL-6 and IFN-γ ELISA kits were from ExCell Bio company (Shanghai, China). Silver stain assay kit was from Beyotime Institute of Biotechnology (Jiangsu, China). Protein A/G beads were from Biogot Technology Co., Ltd. (Nanjing, Jiangsu, China).

### Cell culture

Murine macrophage cell line (RAW264.7) was purchased from Peking Union Medical College, Cell Bank (Beijing, China) and cultured in DMEM supplemented with 10% fetal bovine serum, penicillin (100 U/mL), and streptomycin (100 μg/mL) in a humidified incubator containing 95% air and 5% CO_2_ at 37 °C.

### MTT assay

The cells were seeded in 48-well culture plates in the density of 5 × 10^4^ cells per well. After treatment, culture medium was exchanged with MTT working solution containing 0.5 mg/mL MTT and incubated for 4 h at 37 °C. The medium was removed and 500 μL of DMSO was added. The absorbance (550 nm) was detected and data were expressed as the mean percentage of absorbance in treated vs. control cells. The value of the control was set at 100%.

### Nitric oxide (NO) assay

NO production was determined from the cell supernatant by NO assay kit based on Griess method. RAW264.7 cells were treated with LPS (1 μg/mL) in absence or presence of FMHM (5, 10 and 20 μM) for 24 h. Then, cell culture supernatants were collected and incubated with Griess reagent (supplied by the kit) in a 1:1 ratio to initiate the chromogenic reaction. The optical density was measured in 96-well plates at 540 nm, and sodium nitrite was used as a standard curve.

### ELISA assay for PGE_2_, TNF-α and IL-6

RAW264.7 cells were treated with LPS for 4 h (TNF-α), 8 h (IL-6) and 24 h (PGE_2_) with or without FMHM. Then, cell culture medium was collected and centrifuged at 4 °C, 16000 rpm for 10 min. The supernatants were then collected and used for detecting TNF-α, IL-6 and PGE_2_ concentrations by ELISA kits.

### Immunocytochemistry assay

RAW264.7 cells were fixed with 4% paraformaldehyde for 20 min followed by permeabilization (0.5% Triton X100 in PBS) and blocking (5% BSA in PBS) for 30 min at room temperature. Then, cells were incubated overnight with primary antibody (1:250) at 4 °C and with a secondary antibody (Alexa Fluor 594, 1:500) for 1 h at room temperature. The cells were further stained with DAPI (5 μg/ml in PBS) for 20 min at 37 °C. Finally, the cells were sealed on cover slips and images were acquired using OLYMPUS IX73 fluorescence microscope (Tokyo, Japan) with excitation/emission wavelengths of 590 nm/617 nm for Alexa Fluor-594 and 360 nm/450 nm for DAPI.

### Chemical synthesis of biotin-labeled FMHM (bio-FMHM)

Biotin (4.1 mmoL), FMHM (4.1 mmoL), N, N-dicyclohexyl carbondimide (DCCI) (4.9 mmoL), and 4-dimethylaminopyridine (DMAP) (0.57 mmoL) were combined in dichloromethane (DCM) (30 mL). The solvent was stirred at room temperature for 96 h. The crude products were separated by preparative TLC (CHCl_3_−MeOH, 95:5, v/v) to yield the Bio-FMHM (5.8 mg). Its structure was characterized by MS and NMR analysis. Bio-FMHM:^1^H NMR (500 MHz, CDCl_3_) *δ*_H_9.65 (s, 1H), 7.24 (d, *J* = 3.5 Hz, 1H), 6.64 (d, *J* = 3.5 Hz, 1H), 6.30 (s, 1H), 5.78 (brs, 1H), 5.71 (s, 1H), 5.46 (s, 1H), 5.23 (s, 2H), 4.55 (m, 1H), 4.35 (dd, *J* = 7.4, 4.7 Hz, 1H), 4.22 (t, *J* = 6.5 Hz, 3H), 3.16 (m, 1H), 2.92 (dd, *J* = 12.8, 4.9 Hz, 1H), 2.77 (d, *J* = 12.8 Hz, 1H), 2.66 (t, *J* = 6.5 Hz, 2H), 2.30 (t, *J* = 7.4 Hz, 2H), 1.69–1.37 (m, 6H). ESI-MS (positive ion mode) *m*/*z* 451.1 [M+H]^+^.

### Immunoprecipitation assay

The direct interaction between FMHM and ubiquitin was assayed by pull-down experiment. Ten mg of recombinant ubiquitin protein was incubated with 10μM biotin, Bio-FMHM, FMHM or 0.2% DMSO as control. After incubation for 2 h at 4 °C, the mixtures were incubated with Avidin-agarose (Pierce, Rockford, IL, USA) and shaken for 1 h at room temperature. Immobilized beads were washed 10 times with washing buffer (50 mM HEPES, 30 mM NaCl, 1 mM EDTA, 2.5 mM EGTA, 0.1% Tween20, Cocktail inhibitor, pH7.5) and further washed 5 times with elution buffer (0.1 M Glycine buffer, pH2.8). The elution buffer was collected and centrifuged with Centricon YM-3 (cut-off 3000 Da) into 100 μL at 4 °C, followed by adding 2 × SDS loading buffer and boiling for 5 min. The sample was separated and detected by Western blotting assay.

### Identification of direct target of FMHM

Biotin-labeled FMHM (Bio-FMHM, 20 μM) was incubated with whole cell lysis (RAW264.7, 500 μg) for 2 h at 4 °C with slight shaking. In competition group, 200 μM of FMHM was also added. Control sample was prepared by incubating only with PBS buffer. Then, 0.5 mL of Avidin-agarose beads (Pierce) was added into each sample and shaken for 1 h at room temperature. The beads were washed 10 times with washing buffer (50 mM HEPES, 30 mM NaCl, 1 mM EDTA, 2.5 mM EGTA, 0.1% Tween20, Cocktail inhibitor, pH7.5) and with elution buffer (0.1 M Glycine buffer, pH2.8) for 5 times. The elution buffer was collected and centrifuged with Centricon YM-3 (cut-off 3000 Da) into 100 μL at 4 °C, followed by adding 2 × SDS loading buffer and boiling for 5 min. The samples were separated and detected by silver staining assay kit (Beyotime, Jiangsu, China). The band was isolated, trypsin-digested and identified by LC/MS/MS analysis. The trypsin-digested samples were first filtered through a 0.22 μm micro-pore membrane and then subjected to liquid chromatography coupled with a LTQ Velos pro mass spectrometer (Thermo Scientific, USA). The Captrap Peptide column (20 μL/min) was used to load the Peptide solution (10 μl), and separation of the analytes were achieved on a RP-C18AQ column (100 μm id × 15 cm, Michrom Bioresources, USA), with a column oven temperature of 35 °C. The electro spray voltage was operated at 1.8 kV.

### Real-time quantitative PCR

Quantitative Real-time PCR (qRT-PCR) was performed using Agilent Technologies Stratagene Mx3005P (USA). Total RNA was isolated using RNA Purification Kit (TianGen, Beijing, China). Total RNA was reverse transcribed at 42 °C for 15 min using Fast Quant RT Super Mix (TianGen, Beijing, China) to obtain cDNA. The cDNA was then diluted 40 times and amplified using Trans Start^®^ Green qPCR Super Mix (Transgen, Beijing, China). GAPDH was taken as the internal control. The program for PCR reactions were: 94 °C for 10 min followed by 40 cycles of 94 °C for 30 s, 54 °C for 30 s and 72 °C for 30 s. The primers for real-time PCR are shown in Table 1. At the end of real-time PCR, the CT value of each reaction was provided and the changes in transcriptional level of target gene normalized to GAPDH were calculated by the following formula: Relative mRNA level of target gene (folds of control) = 2^−ΔΔCT^

### Western blot assay

Western blot assay for the expression of different proteins was carried out following the standard protocols. Total proteins were isolated, separated by SDS–PAGE and transferred to polyvinylidenefluoride (PVDF) membranes by semi-dry transfer system. Then, the membranes were blocked with 5% skim milk powder for 1 h at room temperature and incubated with primary antibodies (1:1000 dilutions, Cell Signaling Technology, USA) overnight at 4 °C followed by incubation with anti-rabbit IgG (HRP-linked secondary antibodies, 1:1000 dilutions, Santa Cruz, USA) at room temperature for 1 h. Finally, the blots were developed using Super Signal West Femto Maximum Sensitivity Substrate (Thermo, USA). The relative optical densities were scanned with Kodak Digital Imaging System (Gel Logic 2200Pro, Kodak, USA).

### *In vitro* E2 ubiquitin-loading assay

The loading of ubiquitin (Ub) to E2 enzyme was assayed by detecting the formation of E2-Ub thioester conjugates in a reaction system (E2-Ubiquitin Conjugation Kit, Abcam, USA). Add assay components (2.5 μL of 2 μM purified recombinant E1 Enzyme-UBE1, 2.5 μL of 50 μM Ub, and 5 μL of 0.5 mg/mL E2 enzymes including UbcH1, UbcH2, UbcH5c, UbcH13/MMS2, 5 μL of Ubiquitinylation Buffer, 1 μL of 50 mM DTT, 2.5 μL of 50 mM MgCl_2_) to 0.5 mL EP tubes in order. Mix tube contents gently and incubate at 37 °C for 60 minutes. Quench assays was performed adding 50 μL of 2 × Non-reducing gel loading buffer. The samples were then detected by Western blot analysis.

### Co-IP assay

After treatment, the cells were scraped and incubated with Co-IP buffer (Beyotime Institute of Biotechnology, Jiangsu, China) adding a commercial protease inhibitor cocktail (Sigma-Aldrich) at 4 °C for 20 min. Then, the lyses were centrifuged at 16,000 rpm; 4 °C for 20 min. Primary antibody (1:100 dilutions) was added into the supernatants (400 μL) and incubated at 4 °C with gentle rocking overnight. Immunocomplexes were incubated with 40 μL protein A-agarose beads (fast flow) with gentle rocking for 6 h at 4 °C, and resuspended in 50 μL SDS sample buffer (2×) followed by boiling for 5 min. Finally, the samples were separated by SDS-PAGE and analyzed using the Western blot assay.

### Detection for ubiquitination of TRAF6 in cells

RAW264.7 and HEK293T cells were transfected with HA-tagged TRAF6 plasmids (HA-TRAF6) using transfectamine TM 2000 reagent (Invitrogen, Carlsbad, CA, USA) for 72 h. Then, the cells were treated with or without FMHM (10 μM) for 1 h and cells were lysed in Co-IP buffer. HA-tagged TRAF6 proteins were obtained by Co-IP technology and the ubiquitination levels of TRAF6 were further detected by Western blot assay using anti-K48-linked ubiquitin and anti-K63-linked ubiquitin antibodies.

### Mice acute endotoxemia model

All procedures involving experimental animals were performed in accordance with the Ethical Guidelines for Animal Care of the Peking University Health Science Center and the experimental protocols were approved by Ethics Committee of Peking University Health Science Center based on <Laboratory Animal-Requirements of Environment and Housing Facilities (GB 14925-2001) >and <Beijing Administration Rule of Laboratory Animal >. Balb/c mice (male, 20–25 g, 8-week-old, n = 10) were from VITAL RIVER Laboratories (Beijing, China), and the license number was SCXK (JING) 2012-0001. After arrival, they were acclimatized to a room with controlled temperature (25 °C), humidity (50 ± 10%) and a 12-h day–night cycle (light on 07:00–19:00 h) for at least 7 days before experiment. Animals were divided into four groups. In group one, the animals received intraperitoneal injection of 0.9% sodium chloride solution; in group two, the animals received intraperitoneal injection of LPS (1 mg/kg); in group three, the animals received intraperitoneal injection of FMHM (10 mg/kg) for 1 h, then followed with intraperitoneal injection of LPS (1 mg/kg); and in group four, the animals received intraperitoneal injection of dexamethasone (10 mg/kg, positive control) for 1 h, then followed with intraperitoneal injection of LPS (1 mg/kg). After 3 h of LPS injection, the animals were anesthetized and then sacrificed. The bloods were collected and serum was isolated for further biochemical assay. Moreover, lungs and intestines were removed and hematoxylin-eosin staining (HE) assay was performed. For survival curve analysis, animals were not anesthetized and sacrificed after LPS injection. Animals were continuously observed and the number of surviving mice was documented daily for 10 days. Then, the survival curve was drawn with software GraphPad Prism 5.

### Mice ear swelling model

Balb/c mice (male, 20–25 g, 8-week-old, n = 10) were divided into four groups. In group one, animals did not receive drug intervention; in group two, the animals received intraperitoneal injection of 0.9% sodium chloride solution; in group three, the animals received intraperitoneal injection of FMHM (10 mg/kg); and in group four, the animals received intraperitoneal injection of dexamethasone (10 mg/kg, positive control). One hour after injection, xylene was painted on one ear of each animal except for control group. After 30 min, all animals were anesthetized and sacrificed. The thickness of each pair of ears was measured with vernier caliper. The difference values of each pair of ears were calculated.

### Statistical analysis

Statistical data were expressed as means ± standard deviation (S.D.) in the text and figures. The data were analyzed by one-way analysis of variance (ANOVA). The values depicting *P* < 0.05 were considered as statistically significant.

## Additional Information

**How to cite this article**: Zeng, K.-W. *et al.* Natural small molecule FMHM inhibits lipopolysaccharide-induced inflammatory response by promoting TRAF6 degradation via K48-linked polyubiquitination. *Sci. Rep.* **5**, 14715; doi: 10.1038/srep14715 (2015).

## Supplementary Material

Supplementary Information

## Figures and Tables

**Table 1:** Primer pairs for real-time PCR.

Gene	Sequence
MCP-1	F: 5′-CTTCTGGGCCTGCTGTTCACAGTT-3′
R: 5′-TTCTTGGGGTCAGCACAGACCTCT-3′	
IL-1β	F: 5′-TGGAGAAGCTGTGGCAGCTACCT-3′
R: 5′-GAACGTCACACACCAGCAGGTT-3′	
TRAF6	F: 5′-TTCCAGAAGTGCCAGGTTAATAC-3′
R: 5′-CAAGTGTCGTGCCAAGTGAT-3′	
GAPDH	F: 5′-GGTGAAGGTCGGTGTGAACG-3′
R: 5′-CTCGCTCCTGGAAGATGGTG-3′	
