# Supplementary material for: Natural small molecule FMHM inhibits lipopolysaccharide-induced inflammatory response by promoting TRAF6 degradation via K48-linked polyubiquitination
Source: Sci Rep. 2015 Oct 1;5:14715. doi: 10.1038/srep14715 (PMC4589686; doi:10.1038/srep14715)
Supplement: Supplementary Information [file srep14715-s1.pdf]

Supporting Information

Natural small molecule FMHM inhibits lipopolysaccharide-induced inflammatory response by promoting TRAF6 degradation via K48-linked polyubiquitination

Ke-Wu Zeng[1], Li-Xi Liao[1], Hai-Ning Lv[1], Jiao-Fang Song[2], Qian Yu[2], Xin Dong[1], Jun Li[3], Yong Jiang[1], Peng-Fei Tu[1,*]

[1]State Key Laboratory of Natural and Biomimetic Drugs, School of Pharmaceutical Sciences, Peking University, Beijing 100191, China

[2]Research Studio of Integration of Traditional and Western Medicine, First Hospital, Peking University, Beijing 100034, China

[3]Modern Research Center for Traditional Chinese Medicine, Beijing University of Chinese Medicine, Beijing 100029, China

Figure S1

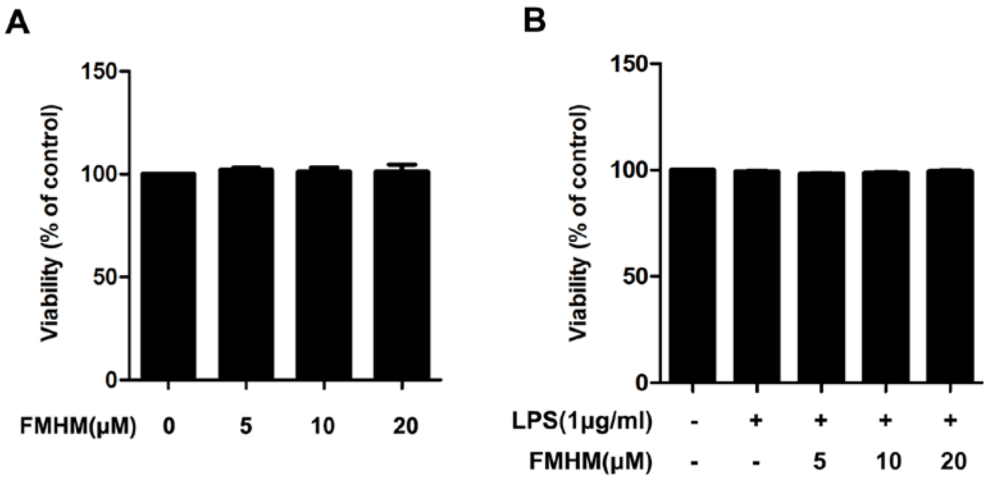
A
B
Viability (% of control)
150
100
50
0
FMHM(μM)
0
5
10
20
LPS(1μg/ml)
-
+
+
+
+
FMHM(μM)
-
-
5
10
20

Figure S2

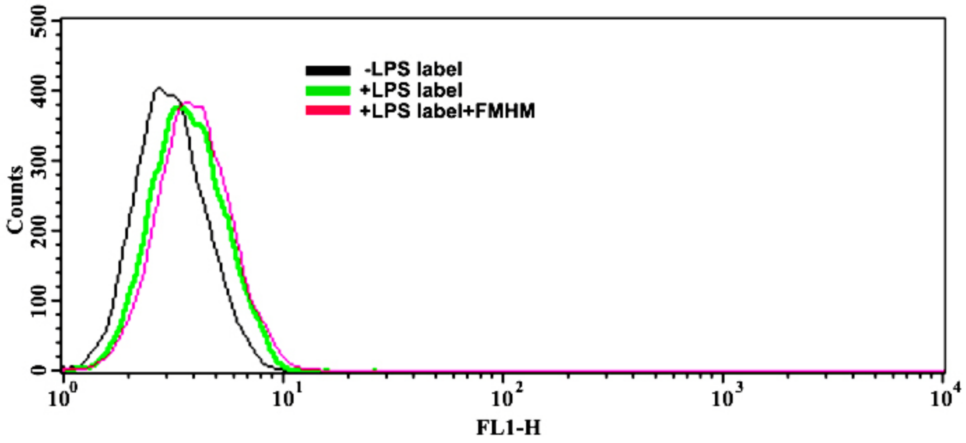
-LPS label
+LPS label
+LPS label+FMHM
Counts
0
100
200
300
400
500
FL1-H
10^0
10^1
10^2
10^3
10^4

Figure S3

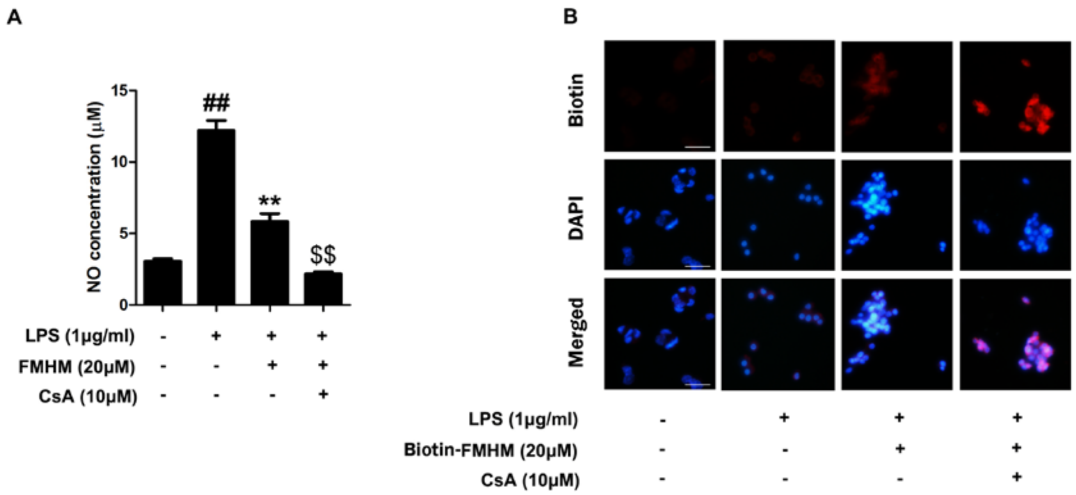
A
NO concentration (µM)
15
10
5
0
##
**
$$
LPS (1µg/ml) - + + +
FMHM (20µM) - - + +
CsA (10µM) - - - +
B
Biotin
DAPI
Merged
LPS (1µg/ml) - + + +
Biotin-FMHM (20µM) - - + +
CsA (10µM) - - - +

Figure S4

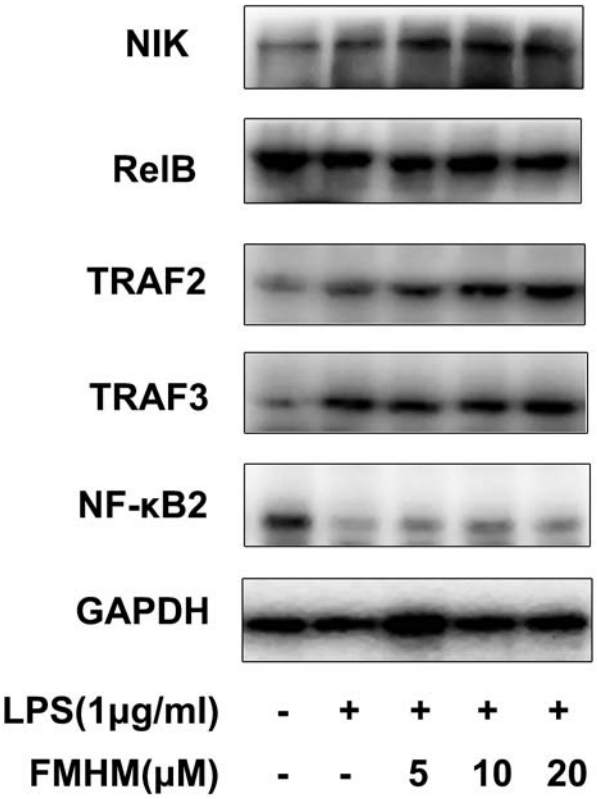

Figure S5

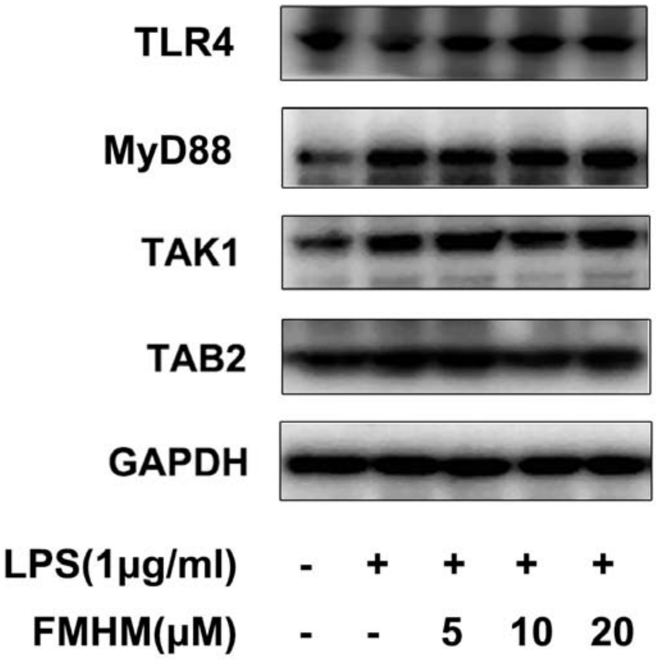

Figure S6

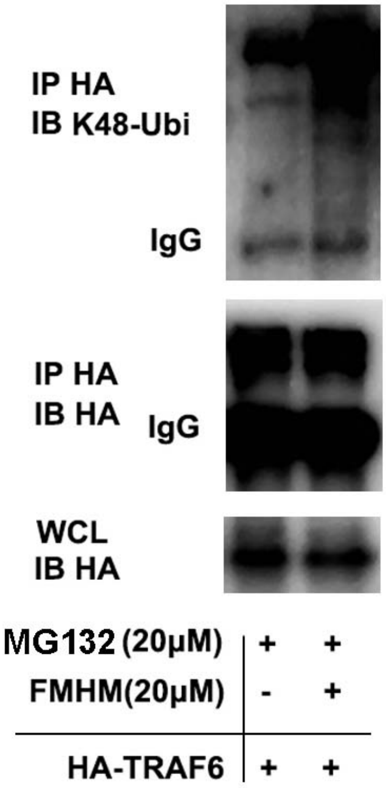
IP HA
IB K48-Ubi
IgG
IP HA
IB HA
IgG
WCL
IB HA
MG132 (20μM) + +
FMHM(20μM) - +
HA-TRAF6 + +

Figure S7

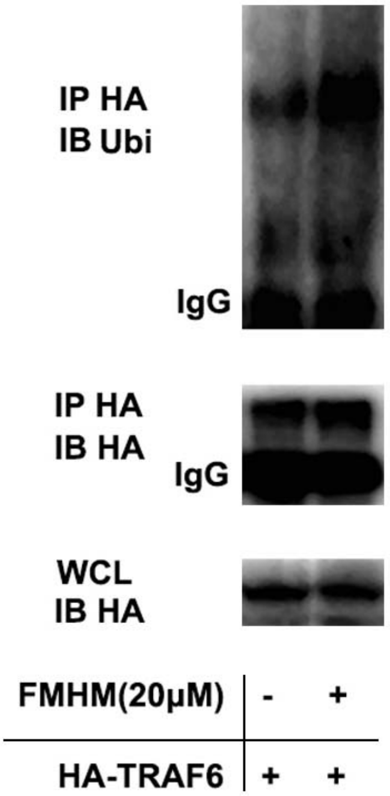

Figure S8

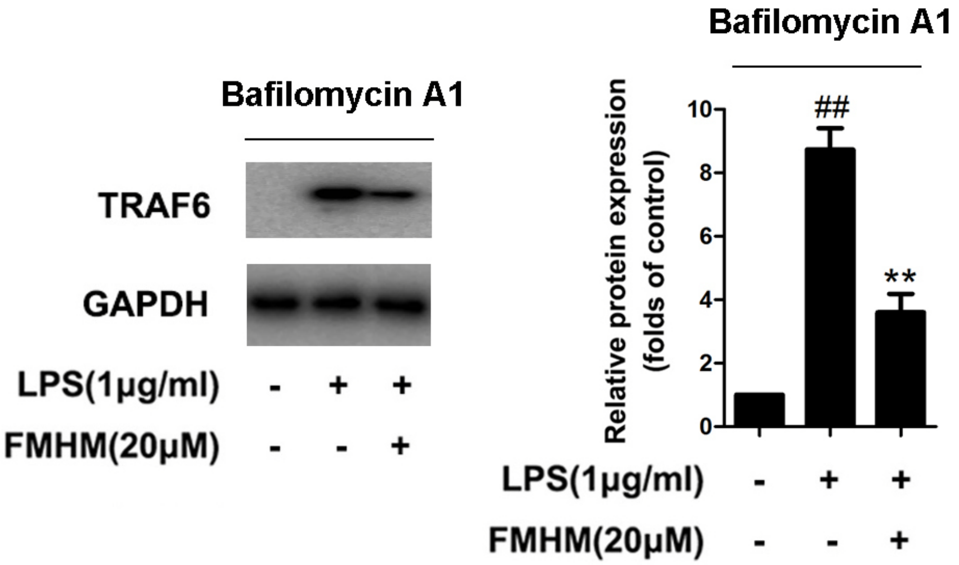
Bafilomycin A1
TRAF6
GAPDH
LPS(1μg/ml) - + +
FMHM(20μM) - - +
Bafilomycin A1
Relative protein expression (folds of control)
10
8
6
4
2
0
##
**
LPS(1μg/ml) - + +
FMHM(20μM) - - +

Figure legends

Supplement Figure S1. FMHM did not show cytotoxicity in RAW264.7 cells. (A) RAW264.7 cells were treated with FMHM (5, 10 and 20 μM) for 24 h, and then MTT assay was done for viability assay. (B) RAW264.7 cells were treated with FMHM (5, 10 and 20 μM) in the presence of LPS (1μg/mL) for 24 h, and then MTT assay was done for viability assay. All data are presented as means±S.D. from independent experiments performed in triplicate.

Supplement Figure S2. FMHM did not interfere LPS-binding to cell surface. RAW264.7 cells were treated with Alexa Fluor488 conjugate LPS (5μg/mL) with or without FMHM (20μM) for 1 h. Then mean fluorescence intensity of 10,000 cells for each sample was quantified by flow cytometry. All experiments were performed in triplicate.

Supplement Figure S3. (A) Ciclosporin A (CsA) increased the NO inhibitory effects of FMHM. RAW264.7 cells were treated with LPS (1 μg/mL) in the absence or presence of FMHM (20 μM) and CsA (5 μM) for 24 h, and then NO assay was performed. (B) RAW264.7 cells were treated with Biotin-FMHM (20 μM) and LPS (1 μg/mL) in the presence or the absence of CsA (5 μM). Then, immunofluorescence assay using anti-biotin antibody (red fluorescence, for FMHM positioning in cells) and DAPI (blue fluorescence, for nuclear positioning in cells) was performed (bar=50μm). All data are presented as means±S.D. from independent experiments performed in triplicate. $^{\#\#}P< 0.01$, relative to control group; $^{**}P< 0.01$, relative to LPS group; $^{\$\$}P< 0.01$, relative to FMHM group.

Supplement Figure S4. FMHM did not regulate non-canonical NF-κB pathway. RAW264.7 cells were treated with LPS (1 μg/mL) in the absence or presence of FMHM (5, 10 and 20 μM) for 24 h, and then Western blotting assay was performed to detect NIK, RelB, TRAF2, TRAF3 and activated NF-κB2 protein. All experiments were performed in triplicate.

Supplement Figure S5. FMHM did not regulate the expressions of TLR4 and adaptor proteins. RAW264.7 cells were treated with LPS (1 μg/mL) in the absence or presence of FMHM (5, 10 and 20 μM) for 24 h, and then Western blot assay was performed for detection of TLR4, MyD88, TAK2 and TAB2 protein expressions. All experiments were performed in triplicate.

Supplement Figure S6. RAW264.7 cells were transfected with HA-tagged TRAF6 plasmids for 72 h, and then treated with MG132 (20 μM) in the presence or absence of FMHM (20 μM) for 1 h. Co-IP assay was performed to detect K48-linked ubiquitination levels of TRAF6. All experiments were performed in triplicate.

Supplement Figure S7. RAW264.7 cells were transfected with HA-tagged TRAF6 plasmids for 72 h, and then treated with FMHM (20 μM) for 1 h. Co-IP assay was performed to detect the overall ubiquitination levels of TRAF6. All experiments were performed in triplicate.

Supplement Figure S8. RAW264.7 cells were pretreated with Bafilomycin A1 (100 nM) for 4 h, and further treated with FMHM (20 μM) in the absence or presence of

LPS (1 μg/mL) for 1 h. Western blotting assay was performed to detect TRAF6 protein. All data are presented as means±S.D. from independent experiments performed in triplicate. $^{##}P < 0.01$, relative to control group; $^{**}P < 0.01$, relative to LPS group.

Primary antibody information

iNOS (No.#2982, CST company); COX-2 (No.#12282, CST company); GAPDH (No.#2118, CST company); K63-Ubi (No.#5621, CST company); K48-Ubi (No.#8081, CST company); Ubi (No.#3933, CST company); TLR4 (No.#14358, CST company); MyD88 (No.#4283, CST company); TAK1 (No.#5206, CST company); TAB2 (No.#3745, CST company); NF-κB Non-canonical Pathway Antibody Sampler Kit #4888 (NIK, RelB, TRAF2, TRAF3 and NF-κB2); NF-κB Pathway Sampler Kit #9936 (p-IKKα/β, IKKβ, p-IκB, IκB, p-NF-κB, NF-κB); HA (No.#3724, CST company); Myc (No.#2278, CST company); TRAF6 (No.EP591Y, Abcam company).
